# Impact of administration of nicorandil prior to percutaneous coronary intervention in treatment of acute myocardial infarction: A protocol for systematic review and meta-analysis

**DOI:** 10.1097/MD.0000000000025565

**Published:** 2021-04-30

**Authors:** Weijun Li, Guozhi Zhang

**Affiliations:** a Department of Cardiovascular Medicine; b Health Management Center, Affiliated Hospital of Xiangnan University, Hunan, China.

**Keywords:** acute myocardial infarction, meta-analysis, nicorandil, percutaneous coronary intervention, protocol

## Abstract

**Background:**

In order to provide new evidence-based medical evidence for clinical treatment, we undertook a systematic review and meta-analysis to assess the efficacy and safety of nicorandil prior to percutaneous coronary intervention in acute myocardial infarction (AMI) patients.

**Methods:**

This systematic review and meta-analysis will be performed according to Preferred Reporting Items for Systematic reviews and Meta-Analyses (PRISMA) guidelines. Two reviewers independently will search randomized controlled trials or observational studies about the treatment of nicorandil on AMI patients. Retrieved databases include Web of Science, ClinicalTrials.gov, Pubmed, Embase, and Cochrane Library. And retrieval time is limited from inception to June 2021. Key words are nicorandil, myocardial infarction, or similar expansion words without publication limitation. Biomechanical studies, in vitro studies, review articles, techniques, case reports, letters to the editor, and editorials are excluded.

**Results:**

The results of our review will be reported strictly following the PRISMA criteria and the review will add to the existing literature by showing compelling evidence and improved guidance in clinic settings.

**OSF registration number:**

10.17605/OSF.IO/UEPKB.

## 1 Introduction

Percutaneous coronary intervention is one of the most effective treatments for the acute myocardial infarction (AMI). However, intraoperative reperfusion injury is not uncommon, presenting as no reflux and recurrent arrhythmia, or chest pain due to reperfusion.^[1]^ The absence of reflux is an independent risk factor for short-term outcomes and long-term cardiac death and events following percutaneous coronary intervention. Patients with this condition may experience increased myocardial infarction size, enlarged left ventricle, decreased cardiac function, malignant arrhythmias, and even death. As a result, the clinical outcome was poor.^[2–4]^

Nicorandil is a K^+^-ATP channel opener with a unique dual mechanism. It can act as a vasodilator similar to nitrate. In addition, it activates K^+^-ATP channels in vascular smooth muscle. The opening of these channels leads to K^+^ outflow and inhibits Ca^2+^ inflow.^[5]^ In this way, calcium overload can be decreased and the occurrence of arrhythmia can be reduced. In addition, small coronary arteries can be dilated and coronary blood flow can be increased.^[3]^ Crucially, nicorandil does not cause adverse reactions, such as sudden drops in blood pressure, bradycardia, and atrioventricular block. Therefore, some investigators have used nicorandil in patients with percutaneous coronary intervention after the absence of reflux.^[6]^

Currently, 2 previous meta-analysis has been published evaluating effect of nicorandil prior to percutaneous coronary intervention on coronary blood flow, cardiac systolic function and clinical outcomes in AMI patients.^[7,8]^ However, these studies have been limited in their ability to provide strong evidences, such as small sample size and inconsistent adherence to modern methodological research standards, making it difficult to draw meaningful conclusions from limited trials. In 2018 and 2020, several new studies were published in the literature.^[9–11]^ Some important information may be obtained if these new original studies are analyzed. Thus, in order to provide new evidence-based medical evidence for clinical treatment, we undertook a meta-analysis to assess the efficacy and safety of nicorandil prior to percutaneous coronary intervention in AMI patients.

## 2 Materials and methods

### 2.1 Searching strategy

This systematic review and meta-analysis will be performed according to Preferred Reporting Items for Systematic reviews and Meta-Analyses (PRISMA) guidelines. The prospective registration has been approved by the Open Science Framework registries (https://osf.io/uepkb), and the registration number is 10.17605/OSF.IO/UEPKB. Two reviewers independently will search randomized controlled trials (RCTs) or observational studies about the treatment of nicorandil on AMI patients. Retrieved databases include Web of Science, ClinicalTrials.gov, PubMed, Embase, and Cochrane Library. And retrieval time is limited from inception to June 2021. Key words are nicorandil, myocardial infarction, or similar expansion words without publication limitation. To minimize the risk of publication bias, we will conduct a comprehensive search that included strategies to find published and unpublished studies. Ethical approval is not necessary because the present meta-analysis will be performed based on previously published studies.

### 2.2 Eligibility criteria

Study included in this review have to meet all of the following inclusion criteria in the PICOS order:

1. population: patients with AMI treatment after percutaneous coronary intervention;
2. intervention group (group 1): nicorandil group;
3. comparison group (group 2): placebo or no nicorandil treatment group;
4. outcome measures: at least one of the following outcome measures was reported: no-reflow phenomenon and corrected thrombolysis in myocardial infarction frame count after percutaneous coronary intervention, wall motion score, left ventricular ejection fraction, risk of heart failure exacerbation of rehospitalization, and incidence of major cardiovascular adverse events during follow-up;
5. study design: RCTs or observational studies.

Biomechanical studies, in vitro studies, review articles, techniques, case reports, letters to the editor, and editorials are excluded.

### 2.3 Data extraction

Two independent authors will extract the following descriptive raw information from the selected studies: study characteristics such as the first author, publication year, study design, follow-up period; patient demographic details such as patients’ number, average age, and gender ratio. The outcomes include no-reflow phenomenon and corrected thrombolysis in myocardial infarction frame count after percutaneous coronary intervention, wall motion score, left ventricular ejection fraction, risk of heart failure exacerbation of rehospitalization, and incidence of major cardiovascular adverse events during follow-up. Where disagreement in the collection of data occurs, this will be resolved through discussion. The corresponding author will be contacted and asked to provide the data if the SD is not reported. In the case of no response, the SD will be calculated from the available data according to the previously validated formula: (higher range value - lower range value)/4 or interquartile range/1.35. The highest SD will be used if the SD cannot be calculated using this approach. If necessary, we will abandon the extraction of incomplete data.

### 2.4 Statistical analysis

The present study will be performed by Review Manager Software (RevMan Version 5.3, The Cochrane Collaboration, Copenhagen, Denmark). Risk ratios with a 95% confidence interval (CI) or mean difference with 95% CI are assessed for dichotomous outcomes or continuous outcomes, respectively. *P* < .05 is set as the level of significance. It will also be considered as statistically significant if “1” is not included in the 95% CI of risk ratios or “0” is not included in the 95% CI of mean difference. The Q test and *I*^*2*^ statistic are used to assess the heterogeneity. When *I*^*2*^ < 40%, it is considered to represent no significant heterogeneity, and then the fixed effect model is used. On contrary, a random effects model is used for the heterogeneity if *I*^*2*^ ≥ 40%. We also conduct the sensitivity analysis to evaluate whether any single study has the weight to skew on the overall estimate and data. The Z test is used to assess the overall effect.

### 2.5 Assessments of study quality

The risk of bias of included RCTs will be independently evaluated by 2 reviewers using the Cochrane risk of bias tool. This tool is employed to assess the quality of RCTs by using following 7 items: random sequence generation, allocation concealment, blinding of participants and personnel, blinding of outcome assessment, incomplete outcome data, selective reporting, and other bias. The Risk of Bias in Non-Randomized Studies of Interventions assessment tool is used to assess the quality of non-RCTs. The Risk of Bias in Non-Randomized Studies of Interventions tool also includes following 7 domains: bias due to confounding, bias in the selection of participants, bias in measurement of interventions, bias due to departures from intended interventions, bias due to missing data, bias in measurement of outcomes, and bias in selection of the reported result. Any controversy will be resolved by discussing with a third reviewer to achieve a final consensus.

## 3 Discussion

Early myocardial reperfusion by primary percutaneous coronary intervention has become an important treatment strategy for patients with AMI, which is associated with reduced infarct size, preserved cardiac function and improved clinical outcomes in these patients. Nicorandil is administered in percutaneous coronary intervention patients by some investigators after the development of the no-reflow phenomenon. Currently, 2 previous meta-analysis has been published evaluating effect of nicorandil prior to percutaneous coronary intervention on coronary blood flow, cardiac systolic function and clinical outcomes in AMI patients.^[7,8]^ However, these studies have been limited in their ability to provide strong evidences, such as small sample size and inconsistent adherence to modern methodological research standards, making it difficult to draw meaningful conclusions from limited trials. In 2018 and 2020, several new studies were published in the literature.^[9–11]^ Some important information may be obtained if these new original studies are analyzed. Thus, in order to provide new evidence-based medical evidence for clinical treatment, we undertook a meta-analysis to assess the efficacy and safety of nicorandil prior to percutaneous coronary intervention in AMI patients.

## Author contributions

**Conceptualization:** Weijun Li.

**Data curation:** Weijun Li, Guozhi Zhang.

**Formal analysis:** Weijun Li, Guozhi Zhang.

**Investigation:** Weijun Li, Guozhi Zhang.

**Methodology:** Weijun Li.

**Resources:** Guozhi Zhang.

**Software:** Weijun Li.

**Supervision:** Guozhi Zhang.

**Visualization:** Weijun Li.

**Writing – original draft:** Weijun Li.

**Writing – review & editing:** Guozhi Zhang.

## References

[1] Feng C Han B Liu Y. Effect of nicorandil administration on myocardial microcirculation during primary percutaneous coronary intervention in patients with acute myocardial infarction. Postepy Kardiol Interwencyjnej 2018;14:26–31. 29743901 10.5114/aic.2018.74352 PMC5939542

[2] Chen C Fu X Li W. Intracoronary administration of anisodamine and nicorandil in individuals undergoing primary percutaneous coronary intervention for acute inferior myocardial infarction: a randomized factorial trial. Exp Ther Med 2015;10:1059–65. 26622439 10.3892/etm.2015.2623 PMC4533183

[3] Hwang J Lee HC Kim BW. Effect on periprocedural myocardial infarction of intra-coronary nicorandil prior to percutaneous coronary intervention in stable and unstable angina. J Cardiol 2013;62:77–81. 23731922 10.1016/j.jjcc.2013.03.017

[4] Lee HC An SG Choi JH. Effect of intra-coronary nicorandil administration prior to reperfusion in acute ST segment elevation myocardial infarction. Circ J 2008;72:1425–9. 18724016 10.1253/circj.cj-08-0212

[5] Ishii H Ichimiya S Kanashiro M. Effect of intravenous nicorandil and preexisting angina pectoris on short- and long-term outcomes in patients with a first ST-segment elevation acute myocardial infarction. Am J Cardiol 2007;99:1203–7. 17478142 10.1016/j.amjcard.2006.12.034

[6] Kurihara H Matsumoto S Tamura R. Clinical outcome of percutaneous coronary intervention with antecedent mutant t-PA administration for acute myocardial infarction. Am Heart J 2004;147:E14. 15077097 10.1016/j.ahj.2003.10.028

[7] Wu M Huang Z Xie H. Nicorandil in patients with acute myocardial infarction undergoing primary percutaneous coronary intervention: a systematic review and meta-analysis. PLoS One 2013;8:e78231. 24167609 10.1371/journal.pone.0078231 PMC3805586

[8] Xu L Wang L Li K. Nicorandil prior to primary percutaneous coronary intervention improves clinical outcomes in patients with acute myocardial infarction: a meta-analysis of randomized controlled trials. Drug Des Devel Ther 2019;13:1389–400. 10.2147/DDDT.S195918 PMC6499143 31118574

[9] Pi SF Liu YW Li T. Effect of sequential nicorandil on myocardial microcirculation and short-term prognosis in acute myocardial infarction patients undergoing coronary intervention. J Thorac Dis 2019;11:744–52. 31019762 10.21037/jtd.2019.02.23 PMC6462716

[10] Feng C Liu Y Wang L. Effects of early intracoronary administration of nicorandil during percutaneous coronary intervention in patients with acute myocardial infarction. Heart Lung Circ 2019;28:858–65. 29891250 10.1016/j.hlc.2018.05.097

[11] Qi Q Niu J Chen T. Intracoronary nicorandil and the prevention of the no reflow phenomenon during primary percutaneous coronary intervention in patients with acute ST-segment elevation myocardial infarction. Med Sci Monit 2018;24:2767–76. 29726480 10.12659/MSM.906815 PMC5954842

